# Modeling and Multiresponse Optimization for Anaerobic Codigestion of Oil Refinery Wastewater and Chicken Manure by Using Artificial Neural Network and the Taguchi Method

**DOI:** 10.1155/2017/2036737

**Published:** 2017-12-26

**Authors:** Esmaeil Mehryar, Weimin Ding, Abbas Hemmat, Muhammad Hassan, Zahir Talha, Jalal Kafashan, Hongying Huang

**Affiliations:** ^1^College of Engineering, Nanjing Agricultural University, Nanjing, Jiangsu 210031, China; ^2^Department of Biosystems Engineering, College of Agriculture, Isfahan University of Technology, Isfahan 84156-83111, Iran; ^3^US-Pakistan Centre for Advanced Studies in Energy, National University of Science and Technology, Islamabad 44000, Pakistan; ^4^Division of Mechatronics, Biostatistics and Sensors (MeBioS), KU Leuven, Kasteelpark Arenberg 30, 3001 Heverlee, Belgium; ^5^Department of Mechanical Engineering in Agromachinery & Mechanisation, Agricultural Engineering Research Institute, Agricultural Research, Education and Extension Organisation, P.O. Box 31585-845, Karaj, Iran; ^6^Institute of Agricultural Resources and Environment, Jiangsu Academy of Agricultural Science, Nanjing, Jiangsu 210014, China

## Abstract

To study the optimum process conditions for pretreatments and anaerobic codigestion of oil refinery wastewater (ORWW) with chicken manure, L_9_ (3^4^) Taguchi's orthogonal array was applied. The biogas production (BGP), biomethane content (BMP), and chemical oxygen demand solubilization (CODS) in stabilization rate were evaluated as the process outputs. The optimum conditions were obtained by using Design Expert software (Version 7.0.0). The results indicated that the optimum conditions could be achieved with 44% ORWW, 36°C temperature, 30 min sonication, and 6% TS in the digester. The optimum BGP, BMP, and CODS removal rates by using the optimum conditions were 294.76 mL/gVS, 151.95 mL/gVS, and 70.22%, respectively, as concluded by the experimental results. In addition, the artificial neural network (ANN) technique was implemented to develop an ANN model for predicting BGP yield and BMP content. The Levenberg-Marquardt algorithm was utilized to train ANN, and the architecture of 9-19-2 for the ANN model was obtained.

## 1. Introduction

Anaerobic digestion (AD) has been utilized by several researchers to produce biogas from organic and inorganic substrates [1–4]. However, during the recent years, the anaerobic codigestion (AcoD) has been suggested by several researchers as being an appropriate technique to improve the performance of the AD process through fulfilling the substrates' inabilities and to provide proper process conditions [4–7]. Given the rich source of aquatic contamination, the oil refinery wastewater is a major challenge for municipal authorities, as the oil refineries are producing bulk quantities of oil refinery wastewater (ORWW) in the world [5, 8]. The AD has been acknowledged as an applicable biological treatment option for the ORWW [5, 8, 9]. The biggest problems, however, with ORWW are the unadaptability of the microorganisms and its containing insufficient nutrients to support the microbial activities [5, 9]. Thus, the AcoD of ORWW, with a rich nitrogen source such as manure, can improve the AD process performance [5]. For example, chicken manure (CM) is one of the well-known substrates to produce biogas through the AD process [1, 7, 10]. Ammonia inhibition or volatile fatty acids (VFAs) accumulation is reported as the unwanted event that occurs during monodigestion of organic substrates [1, 7] which affects the biogas production (BGP) yield negatively. Conversely, the AcoD of different substrates is the appropriate technique to improve their AD performance.

The AD process, like other biological process, contains different complexities. For example, its performance could be affected by several parameters, such as temperature, C/N ratio, feedstock total solids in the AD reactor, type of pretreatment and its conditions, and type of digester [4]. To optimize the pretreating and AcoD process conditions, different optimizing methods were applied [11, 12]. Among them, the Taguchi method is one of the significant methods used to evaluate the different experimental parameters with the lowest number of experiments. In fact, conducting the minimal number of experiments using an orthogonal array to provide the beneficial fermentation about the effect of each factor and introduce the optimum point is the main benefit of Taguchi method [13]. To investigate the effect of several factors on the process performance, the factorial design is one common experimental design; while increasing the number of experimental factors and their levels, the factorial design will be costly and time-consuming [14]. On the contrary, the Taguchi's orthogonal array can investigate the main effect of each factor on the process performance, and it also evaluates the interactions between the factors to obtain the optimum output responses [13]. To optimize the output response, the Taguchi method has been applied in several fields, such as environmental and pollution control, industrial processes, and chemical engineering [11, 13–15]. However, this technique has rarely been utilized in the AcoD process optimization until this point.

Applying mathematical equations, such as modified Gompertz model, transfer function, cone index function, or logistic function to model the cumulative BGP curve, was deeply focused on in previous research efforts [16]. Meanwhile, applying an artificial neural network (ANN) to model the BGP yield based on the initial and kinetic parameters of the AcoD process (such as temperature, pH, total ammonia nitrogen (TAN), total volatile fatty acids (TVFA) and their profile, free ammonia nitrogen (FAN), and CODS) was seldom investigated [12, 17, 18].

The main objective of this research was to obtain the optimum conditions for the AcoD process of ORWW with CM under different pretreating and process conditions. To describe the interactions between experimental factors, the analytical AcoD process parameters including pH, CODS, TVFA and VFAs profile, TAN, and FAN were experimentally measured. An ANN model was also developed for predicting the yield and quality of produced biogas based on experimental data.

## 2. Materials and Methods

### 2.1. Feedstocks and Inoculum Collection and Pretreatment

The ORWW used in the experiments was provided by the Jinlin SINOPEC oil refinery factory, in Nanjing, Jiangsu Province, China. The CM was obtained from a local livestock farm in the Laoshan district of Nanjing, Jiangsu, China. Collected substrates were stored at (4 ± 0.5)°C until their use in AcoD experiments. The anaerobic sludge was collected from an active biogas plant which was working with pig manure from a local livestock farm, which was located in the Laoshan district of Nanjing, Jiangsu, China as well. To remove the dissolved methane and easily degradable organic matter in the collected sludge, the activation stage was conducted according to the method described by Xi et al. [10], and then it was utilized as an inoculum. To pretreat the ORWW, the sonicator (Model-KQ5200DE, China) with an ultrasonic power of 200 W and sonication frequency of 20 kHz at three different time durations (0 min as a control and 15 and 30 min) was used. The chemical characteristics of the pretreated and untreated substrates are presented in Table 1.

### 2.2. BMP Experimental Setup and Procedure

One liter of lab-scaled digesters was utilized to do BMP testing and to evaluate the different process conditions. The applied anaerobic digester mechanism was partially the same as that used by Hassan et al. [19]. All the BMP experiments were conducted in triplicate, and the digestion period was continued until the daily biogas yield was found lower than 1% of the previous total yield. The working volume of each digester was kept at 800 mL, with 400 mL inoculum as an optimized amount of the activated sludge to do codigestion with ORWW [5]. The designing of experiments and substrate compositions was conducted based on Taguchi's L_9_ orthogonal array, which will be discussed in the following sections. The mixtures of CM with seed sludge (Control 1) and ORWW with seed sludge (Control 2) were applied in two experiments for comparing and evaluating the effects of AcoD and pretreatments. To investigate the background biogas production from inoculum, three digesters which contained unmixed inoculum were used as blank assay experiments. And then based on their results, the BGP yields for different experiments were corrected. The daily BGP volume from each digester was recorded by the liquid displacement method, in which the saturated NaHCO_3_ solution was utilized as a displacing liquid [10]. The biogas samples were daily analyzed for composition (N_2_, CH_4_, and CO_2_) using a gas chromatograph (GC 7820A, Agilent, USA) equipped with PQ 80–100 mesh column and thermal conductivity detector (TCD). The temperature of TCD and column were kept at 250°C and 90°C, respectively, while the flow rate of 25 mL/min Helium (He) as the carrier gas was applied.

### 2.3. Analytical Procedures

The chemical characteristics of pretreated and untreated ORWW, CM, and inoculum were obtained prior to the BMP experiments and in accordance with the Standard Methods of American Public Health Association [20]. The chemical oxygen demand solubilization (CODS) was determined every three days, and its stabilization rate for each experimental run was calculated using the following [19]:

(1) $$\begin{array}{c} CODs\left(\;\%\;\right)=\frac{{CODs}_{i}-{CODs}_{f}}{{CODs}_{i}}\times 100, \end{array}$$

where *i* and *f* are the initial and the final CODS values during the AcoD process, respectively. The pH value was directly measured from the liquid samples with a digital pH meter (FE20K, Mettler-Toledo, Switzerland). To survey the total ammonia nitrogen (TAN) and free ammonia nitrogen (FAN), the ammonia meter (Lianhua Tech. Co., China) was used to measure the TAN content in accordance with the method described by Hassan et al. [19]. The FAN content was obtained using the following [3]:

(2) $$\begin{array}{c} FAN=TAN\times {\left(\;1+\frac{10^{-pH}}{10^{-\left(0.09018+2729.92/T\right)}}\;\right)}^{-1}, \end{array}$$

where FAN is the free ammonia nitrogen content (mg/L), TAN is the total ammonia nitrogen content (mg/L), and *T* is the process temperature (Kelvin). The total content of volatile fatty acids (TVFA) and also VFAs profile (including Acetic, Propionic, Butyric, and Valeric acids) were determined by Gas Chromatography (Model GC-2014, Shimadzu, Japan) equipped with thermal conductivity detector (TCD), having column (DA, 30 m × 0.53 mm × 1 *μ*m Stabilwax) and flame ionization detector, while injector and detector temperatures were kept at 150°C and 240°C, respectively.

### 2.4. Optimization Process Conditions Using Taguchi Method

To investigate the effects of different process conditions on the AcoD process performance, four parameters (ORWW portion, process temperature, sonication time, and TS level of feedstock in the digester) were taken into consideration. Using Design Expert software (Version 7.0.0), the orthogonal array L_9_ (3^4^) was applied to design the nine significant experimental runs instead of 81 runs in the case of factorial design for four parameters, every parameter with three levels.  Table 2 indicates the assigned parameters and their levels.  Table 3 presents the experimental design of L_9_ orthogonal array for running AcoD experiments. In the optimization process, achieving desired output values was the main objective. That “signal” (*S*) represents this index in the Taguchi method, while “noise” (*N*) illustrates an undesirable output characteristic [13]. Thus, the* S/N* ratio is the ratio of signal to noise, and the Taguchi method uses this ratio to estimate the quality of experimental outputs in comparison with the desired output values [13]. In other words, the* S/N* ratio indicates the effects of each experimental factor on the output [14]. Chen et al. [13] reported that the* S/N* ratio could be divided into three categories: (i) nominal output is the best (NTB); (ii) smaller output is the best (STB); and (iii) larger output is the best (LTB) [15]. To obtain the maximum BGP, BMP, and CODS stabilization rate, the LTB was applied in this study, and the* S/N* ratio was calculated using the following [13, 15]:

(3) $$\begin{array}{c} \frac{S}{N_{LTB}}=-10\log\left[\;\frac{1}{n}\overset{n}{\underset{i=1}{\sum }}\frac{1}{y_{i}^{2}}\;\right], \end{array}$$

where *S*/*N*_LTB_ is “*S*/*N* ratio for larger output is the best”, *n* is the number of experimental runs for one parameter or output, and *y*_*i*_ is the experimental output, that is, BGP (mL/gVS) or BMP (mL/gVS) or CODS stabilization rate (%) in the present study.

To evaluate the effects of each factor on the outputs, the average *S*/*N* ratio for each factor *n* at level *m* was calculated by applying the following [14]:

(4) $$\begin{array}{c} \frac{S}{N}=\frac{\text{Sum of }S/N\text{ ratios for factor }n\text{ at level }m}{3}. \end{array}$$

The wide range of *S*/*N* ratios of different levels for each factor illustrated the higher effect of that factor on the outputs. The results of *S*/*N* ratios variation range and factors ranking are presented in Table 4.

### 2.5. Modeling Using Artificial Neural Network (ANN)

To develop a model for predicting biogas production and its quality based on the AcoD kinetic parameters, the artificial neural network (ANN) modeling was utilized by the assistance of Neural Network Toolbox of MATLAB 8.1.0 (R2013a) software. The simulation was conducted by using a notebook with 2.59 GHz Intel (R) Core (TM) i7 processor and 12.00 GB RAM. In the purposed ANN, nine different AcoD kinetic parameters including TAN, FAN, TVFA, Acetic acid, Propionic acid, Butyric acid, Valeric acid, temperature, and pH were assumed as the ANN inputs and the BGP yield (mL/gVS) and its BMP content (%) were assumed as the ANN outputs.

Designing ANN architecture is one of the main steps to develop an ANN model. The ANN architecture contains three layers which are as follows: (i) an input layer contains input neurons. The number of input neurons is equal to the number of network inputs, which are nine in this research. (ii) A hidden layer includes several hidden neurons which transform the inputs to targets. Designing this layer and choosing the right numbers of hidden layer are an important task of developing ANN model [21]. The simulation was started from 5 neurons in a hidden layer and was increased by an interval of 5. Then the best architecture performance based on the mean square error (MSE) and correlation coefficient (*R*) was obtained. The results of this step are presented in Table 6; (iii) an output layer includes output neurons which are predicted values for process response. As stated previously, the BGP yield (mL/gVS) and its BMP content (%) are the ANN outputs in the present study. The experimental data set including inputs and outputs was randomly divided into three sets. In other words, 70%, 15%, and 15% data sets were applied as the training set, validation set, and testing set, respectively. The Levenberg-Marquardt (LM) algorithm was run as the training algorithm. To estimate the ANN model prediction accuracy, the MSE (see (5)) and regression coefficient function (see (6)) were calculated for predicted outputs [18]:

(5) MSE=1n∑i=1ny^i−yi2,

(6) R2=1−∑i=1nyi−y^i2∑i=1nyi−ym2,

where ${\hat{y}}_{i}$ is the predicted output value, *y*_*i*_ is the observed output value, *y*_*m*_ is the mean of observed output values, and *n* is the total number of samples in the data set, respectively.

## 3. Results and Discussion

### 3.1. Biogas Production and AcoD Process Parameters

**Figure 1:**
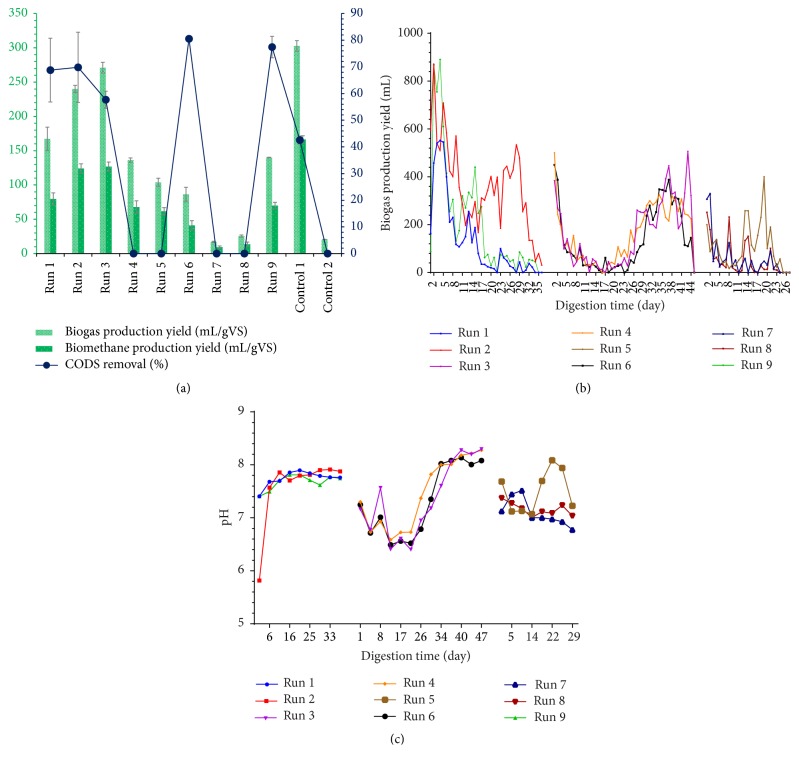
(a) Cumulative biogas and biomethane production and CODS stabilization rates, (b) daily biogas production yields, and (c) pH variation trends for different experimental runs.

**Figure 2:**
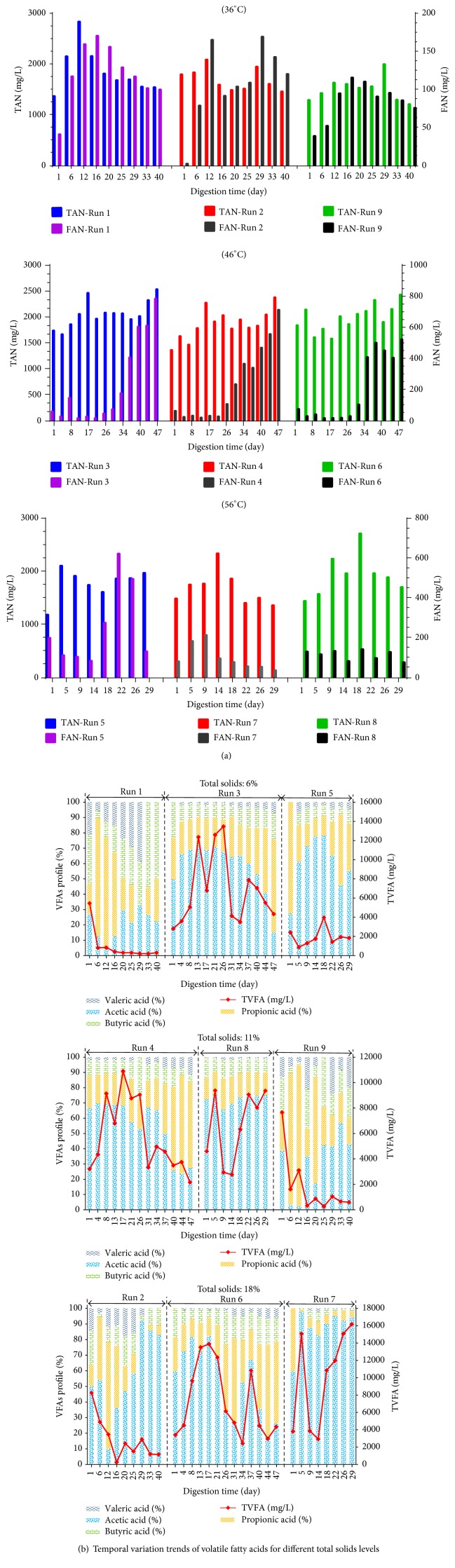


The experimental results of the daily biogas production yield and cumulative BGP and BMP for the different experimental runs and controls are shown in Figures 1(a) and 1(b) and Table 3. The achieved results confirmed the propagation of AcoD of ORWW with CM, which was in accordance with previous research [5]. The negligible BGP and BMP by ORWW monodigestion was also observed; this revealed that the AD conditions for supporting microbial activities were inappropriate. In contrast, Control 1 digester produced the largest yield of BGP and BMP among all conducted experiments. However, focusing on the optimizing experimental runs (Run 1 to Run 9) to investigate the optimum process conditions was the main objective of the present study. The experimental results illustrated that the digesters of the Run 3 test produced the highest cumulative BGP and BMP yields among experimental runs, which contained 44% treated ORWW with 0 min sonication time and 6% TS level in the 46°C condition. Because of their TS levels and CM ratios in the AD digester, some digesters such as Run 7 and Run 8 contained a high level of organic matter (OM). Low BGP yields and CODS stabilization rate, though, confirmed that these treatments have unacceptable AcoD process performances. The value of CODS stabilization rate for some of the experimental runs was less than zero, which was due to the AD inhibition and hydrolyzed organic components accumulating in the digesters. These values were assumed zero in Figure 1(a), while their values are reported in Table 3. The AcoD process is a multistep, complex process; its performance can be affected by several parameters including temperature, substrates characteristics, C/N ratio, TAN, FAN, pH, VFAs profile, and trace elements [22]. The temporal variation trends of pH, TAN, and FAN for different experimental runs were shown in Figures 1(c) and 2(a). The distributions of individual VFAs (Acetic, Propionic, Butyric, and Valeric acids) are shown in Figure 2(b). Focusing on their variation trends for each run could be helpful in finding the optimum conditions.

#### 3.1.1. Run 1, Run 2, and Run 9 (Process Temperature of 36°C)

The estimated results concluded that the TAN and FAN concentrations for these three runs were in the suitable range. Also, the TVFA concentration and pH variation proved that these runs had a successful and stable fermentation processes. Generally speaking, the wide ranges of 1700 mg/L to 14000 mg/L for TAN and 200 mg/L to 800 mg/L for FAN were reported as undesirable ranges for successful AD [2, 6]. Their unsimilarity, which led to wide ranges of TAN and FAN, was related to the differences in the type of substrates, pH, temperature, digester type, and TS level. Thus, focusing on the TAN and FAN parameters in addition to discussing other process parameters such as VFAs profile and pH variation trend can be more beneficial. Digester Run 2 produced the second highest BGP yield among all of the runs. This digester contained more CM ratio because of TS level of 18%. However, a higher concentration of Propionic acid in comparison with Acetic acid was also observed. Additionally, a high concentration of Acetic acid proved an unhealthy AD system for this digester. Kwietniewska and Tys [23] demonstrated that if the ratio of Propionic acid to Acetic acid was >1.4 and the Acetic acid concentration was >800 mg/L, a signal of digester failure can be observed. With regard to the level of TS (18%) and AcoD ratio of CM in the digester (55%), it may be noted that the sufficient OM was available in this digester that was responsible for the stable AD system, while, by comparing the BGP values of Run 1, Run 2, and Run 9 from the point of utilized CM amount (as a rich organic source and well-digestible substrate), Run 2 had produced the lowest BGP yield. It may be noted that the lower TS can be preferred. In comparing Run 9 with Run 5, Run 9 contained higher OM (because of TS level of 11%). However, lower temperature led to lower FAN and TAN concentrations and provided anoxic conditions for continuation of the AD process. Furthermore, the variation trends of pH and individual VFAs profile proved that the produced VFAs were consumed during the digestion period and prevented the VFAs accumulation. The CODS stabilization rate of this experiment was estimated to be (77.44 ± 4.03)%, which illustrates its acceptable substrates biodegradation performance.

#### 3.1.2. Run 3, Run 4, and Run 6 (Process Temperature of 46°C)

During the initial days of the digestion period, noncritical values of TAN and FAN for digesters Run 3, Run 4, and Run 6 were observed. While continuing the AcoD process, their FAN concentrations were increased to the level of 700 mg/L, which resulted in the AD inhibition. Rajagopal et al. [3] had reported that the FAN ranged above 250 mg/L with 10% of TS at 35°C is toxic for the anaerobic fermentation of CM. Moreover, the FAN concentrations were found to be more than 700 mg/L at the thermophilic temperature (55–65°C), which could lead the AD to inhibition and rapid increase in the VFAs concentrations (>5000 mg/L) [3]. Although the TVFA concentration was found sequentially higher than 10000 mg/L, the pH variation trends and BGP yield confirmed the acceptable fermentation process performance. With reference to the digester Run 4 and its low BGP yield, its FAN could be mentioned as an inappropriate affecting index. Chen et al. [6] reported that releasing levels of TAN and FAN concentrations were the results of AD of livestock, which affected the methanogenic bacteria activities and led to low methane production. Also, the TVFA concentration of this run was ranged above 10000 mg/L, which had confirmed the AD inhibition. However, availability of sufficient CM (TS of 11% with AcoD ratio of 66%) had provided adaptability conditions for the microorganisms. The pH variation range for the last weeks of the digestion period illustrated that these conditions caused the AD system to be stable with remarkable BGP. Meanwhile, focusing on the CODS removal rate demonstrates that the AD process performance of this run was unacceptable. The AD process is a biological gasification system which consumes higher organic substrates and converts their components to methane-rich biogas that leads to consuming substrate COD. Hence, the CODS stabilization rate is considered as one of the most important parameters in case of AcoD of wastewater with some other substrates. The general principles of AD process for digester Run 6 were similar to Run 3 and Run 4, but the TS level of 18% had provided enough CM to geminate the FAN and TVFA production and push them to the undesirable ranges which had decreased the AD process performance. By focusing on the pH variation trend (Figure 1(c)), the unbeneficial effects of the pH increasing on the FAN concentration could be proven. Chen et al. [6] had reported that the pH increasing from 7 to 8 could lead to eight times increase in FAN concentration; thus, the phenomenon leads to an increased TAN and FAN inhibitory effect on the AD process.

#### 3.1.3. Run 5, Run 7, and Run 8 (Process Temperature of 56°C)

Among all runs, the lowest TS level (6%) and AcoD ratio of CM (44%) were applied in digester Run 5. However, its FAN concentration had increased to above 600 mg/L, which leads to the AD inhibition. In general, the FAN was mainly a function of the TAN concentration, temperature, and pH [6]. Rajagopal et al. [3] reported that increasing the metabolic rate of microorganisms and FAN concentration can result in AD temperature increase. On the other hand, they stated that the inhibitory effect of unionized ammonia form (FAN) is more than the ammonium ion (NH_4_^+^) in high pH values [3]. Generally, although the individual VFAs profiles illustrated healthy AD systems, the value of (−81.33 ± 285.72)% for CODS stabilization rate confirmed that the AcoD process performance for this run was unacceptable. Based on Table 3, the highest level of TS (18%) and AcoD ratio for CM (66%) was applied to digester Run 7, and the highest amount of CM (as high as it contained in OM substrate) was fed to this digester. However, the lowest BGP yield was observed. Based on the previous studies and above discussed results, the high level of C/N ratio and the high sensitivity of the AD process to the FAN concentration in the thermophilic conditions could be noted as the main reasons for this observation [2, 3, 6]. Wang et al. [24] demonstrated that one of the most important and crucial parameters in the AD process was the C/N ratio, which its low values led to the ammonia accumulation and resulted in AD inhibition [24]. Of course, Zhang et al. [2] noted that the high ammonia concentration was an unsatisfactory reason for the AD system failure and even any reduction in BGP. The high TVFA concentration, though, could be observed through experimental results which were more than 15000 mg/L at the end of the digestion period. The VFAs accumulation was reported as one of the main problems in the AD process of CM and other types of dairy and poultry manures [1, 25]. Ahn et al. [25] showed that VFAs concentration above 12000 mg/L results in the pH reduction which also inhibited the methanogenic activities for converting the VFAs into methane [25]. Similar to Run 7, the VFAs accumulation in digester Run 8 caused a decrease in the AD process performance and illustrated the second lowest BGP yield among experimental runs. In fact, the presence of CM and enough TAN (>2000 mg/L) caused the biogas production in the digesters, which is in line with the previous studies [1]. From the point of biodegradation efficiency, the negative values of the CODS stabilization rate for these two runs were observed, confirming their unacceptability during the AD process performance.

### 3.2. Optimization of AcoD Process Conditions

**Figure 3:**
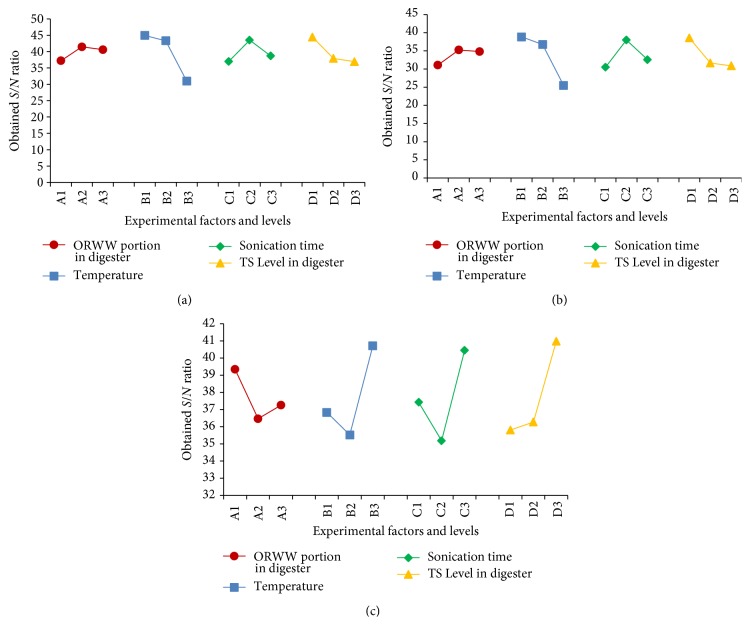
*S/N* responses for different formulations for (a) biogas production yield, (b) biomethane production yield, and (c) CODS removal rate.

Taguchi's L_9_ (3^4^) orthogonal array was applied to estimate the optimal AcoD process conditions for ORWW with CM, which had provided the maximum BGP yield with the highest quality (BMP content) and maximum CODS stabilization rate. As introduced in the “test procedure” section, four control factors were ORWW portion in digester (%) as “A,” temperature (°C) as “B,” sonication time (min) as “C,” and TS level in digester (%) as “D.” To obtain the effects of each factor on the response and estimate the optimal conditions, calculating* S/N* ratios for different levels of each factor by using (4) was found to be useful [11, 13, 14]; their values are represented in Table 4 and Figure 3. The analyzed experimental results confirmed that the process temperature had the largest influence on the BGP and BMP yields, while the sonication time played the most effective role on CODS removal rate. For BGP and BMP yields, the effects of substrate TS level in the digester were more influential than the sonication time. Conversely, the effect of temperature was more important than the effect of TS level on CODS removal rate. However, the estimated values for* S/N* ratios indicated that the AcoD ratio (ORWW portion in digester) had the most neglected important effect on the experimental responses. Among the AcoD process parameters, the FAN was the most sensitive parameter to the temperature increase [3], which could be considered the main reason for the low performance of the AD process of several of the experimental runs. On the other hand, Abouelenien et al. [1] showed that the temperature had a significant effect on the VFAs production during AD process of CM. Therefore, high ammonia concentration and VFAs at the same time can lead to the AD process failure and a decrease in the BGP yield [2]. As the second most effective control factor on the experimental results, the high level of TS can lead to a bigger amount of OM in the digester and then higher VFAs production, which affected the AD process performance. Generally speaking, focusing on the BGP yields of experimental runs and Figure 2 (TAN, FAN, and VFAs profiles) indicated the same results. Based on the experimental responses, which were presented in Table 3, the formulation of A2B2C3D1 (Run 3) generated the maximum BGP and BMP yields, 44% nontreated ORWW with process temperature of 46°C and TS level of 6% in digester. Under such process conditions, the values of (270.99 ± 7.87) mL/gVS and (126.79 ± 6.74) mL/gVS were obtained as the cumulative BGP and BMP yields, respectively, while the rate of (80.57 ± 0.40)% as the highest CODS stabilization rate was observed for the formulation of A3B2C1D3 (Run 6). The estimated* S/N* ratios for different levels of each factor (Table 4 and Figure 3) indicated the different formulations as the optimal conditions.

Figure 3 illustrated that the formulation of A2B1C2D1 was the optimal condition for the maximum BGP and BMP yields. Also, it shows that the formulation of A1B3C3D3 contained the optimal conditions to perform the maximum CODS removal rate. Conversely, the numerical optimization results which were obtained by using Design Expert software (Version 7.0.0) estimated another formulation as having the optimal conditions. The main difference between this formulation and the results of last two methods is multiresponse optimizing and mentioning three responses at the same time. In fact, the software mentioned three responses and indicated one formulation, while, in the last two methods, each response was separately focused and their* S/N* ratios were separately calculated and compared. Furthermore, a considerable error could happen by the minus values of CODS removal rate, which present a bigger* S/N*, based on (3). On the other hand, checking the results of solutions for combinations of categorical factor levels illustrated that the negative effect of minus values for CODS removal rate was mentioned by software. And the optimum point was investigated. However, because of the variability of the true optimal factor levels, their values could not be guaranteed by using orthogonal design, and they may be different from the corresponding predetermined factor levels [11]. Therefore, to verify the results and formulation of optimal conditions, the experiment of the A2B1C2D1 formulation was conducted in three replicates that was the estimated formulation of optimal conditions suggested by the software. The experimental responses of the suggested formulation, which are shown in Table 5, consequently proved the optimal conditions obtained by this formulation. In summary, the optimal conditions were 44% ORWW portion, process temperature of 36°C, 30 min for sonication time, and 6% of TS level of substrates in the digester.

The observed results showed that the AcoD of ORWW with CM was successful. And in comparing with the results of Control 1, almost same BGP and BMP were observed by applying 44% lower CM (as a valuable substrate). Also, the CODS stabilization rate for optimum condition was increased by more than 50%, which confirms the improving substrates biodegradability in the digester. It may be noted that the desirability of A2B2C2D1 was 1.000, the same as desirability of examined conditions. But, lower energy consumption to provide the temperature of 36°C made the process conditions of A2B1C2D1 more logical.

### 3.3. Artificial Neural Network (ANN) Modeling of Biogas Production

**Figure 4:**
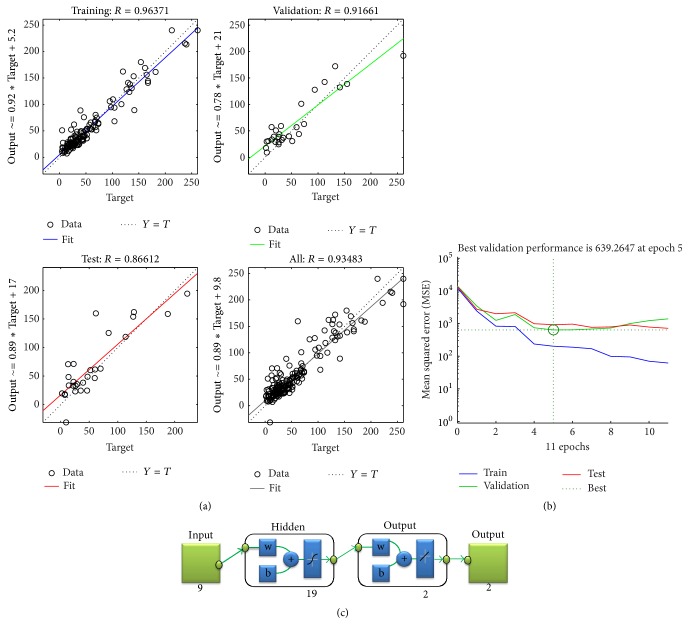
ANN modeling results: (a) regression plot, (b) performance, and (c) ANN model.

**Figure 5:**
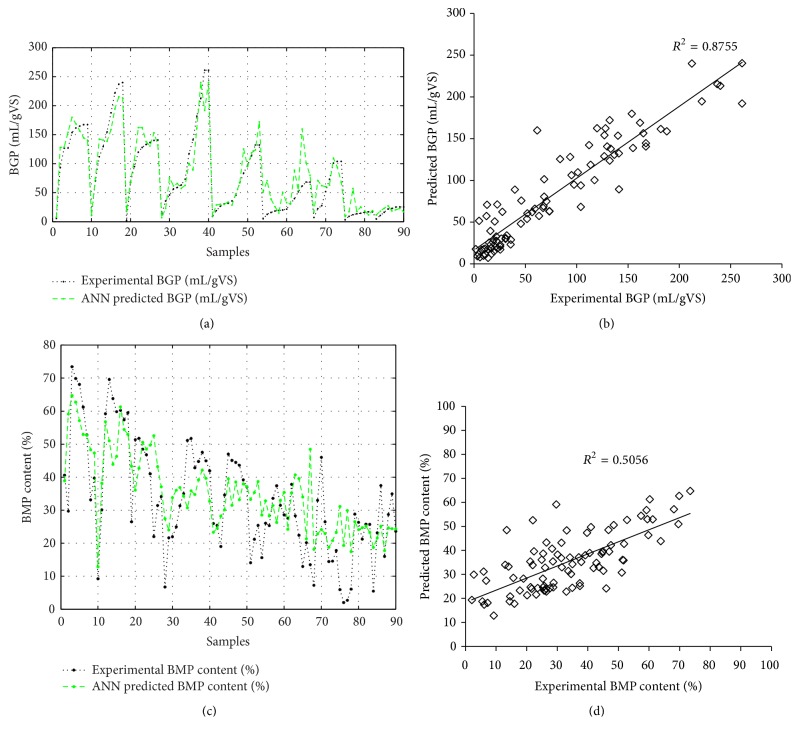
(a) Experimental and model predicted BGP yield (mL/gVS), (b) validation ANN model for predicting BGP yield, (c) experimental and model predicted BMP content (%), and (d) validation ANN model for predicting BMP content.

Implementing the experimental AcoD process parameters values, including TAN, FAN, TVFA, Acetic acid, Propionic acid, Butyric acid, Valeric acid, temperature, and pH as inputs, an ANN model was developed. To obtain the right architecture for the proposed ANN, the number of hidden neurons was optimized, and the results are shown in Table 6. As illustrated, the MSE value for 50 and 30 neurons had very low values, while their MSE values for validation and test sets were very high. This resulted in overfitting and obtaining unacceptable correlation coefficient (*R*) values for validation and test sets. It may be noted that the small difference between experimental results led to big squared error [18]. The high values for the obtained MSE values are stated in Table 6. Checking MSE and* R* values for other numbers of hidden neurons demonstrates that the right choice should be selected between 15 and 20 neurons in the hidden layers. Between them, the lowest values of MSE for training, validation, and testing were 187.2529, 519.463, and 919.5157 for 18, 17, and 19 neurons in the hidden layer, respectively. From this, the values of correlation coefficient (*R*) show that the results of 19 neurons in the hidden layer is the appropriate design (the architecture of 9-19-2) for the proposed ANN model. The simulating method and evaluating results are in line with previous research [21].  Figure 4(b) demonstrates the ANN training performance. Maximum 1000 epochs were assumed in the ANN designing step. However, the best validation performance, 639.2647, was obtained at epoch 5. The results of prediction accuracy (MSE, *R*, and *R*^2^) showed that the ANN model with 9-19-2 architecture provides a significant relationship between input and output parameters. Figures 5(a) and 5(c) illustrate the experimental and predicted values for BGP yield and its BMP content. Their *R*^2^ are shown in Figures 5(b) and 5(d), which shows the prediction accuracy of ANN outputs separately.

The estimated *R*^2^ for BGP yield and its BMP content were 0.8755 and 0.5056, respectively. It could be noted that the designed ANN model for predicting BGP yield was accurate, while its prediction for the BMP content of produced biogas was imprecise. As the reason for this observation, Nair et al. [17] reported that the biological process contained several complexities and resulted in the process which is dependent on the different process conditions: microorganism behavior, type of reactor, and so forth [17]. Therefore, to improve ANN model accuracy, increasing the number of data sets and/or focusing on more analytical parameters of AcoD process as the ANN inputs could be beneficial.

## 4. Conclusion

The results of experimental runs revealed the highest BGP and BMP yields were observed for Run 3. Meanwhile, the highest CODS rate was performed for Run 6. However, multiresponse optimization showed the optimum conditions of 44% ORWW, 36°C temperature, 30 min sonication, and 6% TS conditions. In comparison to CM monodigestion, the optimum conditions provided similar BGP and BMP volumes with utilizing 44% less CM and 64.95% higher CODS rate. Besides, the ANN model by the architecture of 9-19-2 was developed to predict the BGP yield and biomethane content and *R*^2^ of 0.8755 and 0.5056 was obtained, respectively.

## Figures and Tables

**Table 1:** Chemical characteristics of the utilized substrates.

Characteristics	Substrates				
	Sonicated ORWW with different time durations			CM	Inoculum
	0 min	15 min	30 min		
TS (%)	1.73 ± 0.03^*∗*^	1.81 ± 0.01	1.94 ± 0.06	33.86 ± 2.50	2.51 ± 0.10
VS (% of TS)	2.69 ± 0.70	2.82 ± 0.45	2.77 ± 0.23	65.35 ± 0.60	49.35 ± 1.00
TOC (g/Kg of TS)	30.28 ± 2.10	31.14 ± 1.89	31.09 ± 0.51	440.22 ± 3.50	36.81 ± 4.30
OM (g/Kg of TS)	52.17 ± 2.95	53.65 ± 2.66	53.57 ± 0.72	758.49 ± 4.94	63.42 ± 6.05
TN (g/Kg of TS)	0.20 ± 0.01	0.19 ± 0.03	0.21 ± 0.12	42.27 ± 0.10	2.04 ± 0.20
C : N ratio	151.40 : 1	163.89 : 1	148.05 : 1	10.41 : 1	17.71 : 1

^*∗*^Mean ± Stdev.

**Table 2:** Experimental parameters with levels.

Parameters	Levels		
	Level 1	Level 2	Level 3
A: ORWW^*∗*^ portion in digester (%)	33	44	55
B: temperature (°C)	36	46	56
C: sonication time (min)	15	30	0
D: total solids level in digester (%)	6	11	18

^*∗*^Oil refinery wastewater.

**Table 3:** Design of Taguchi's L_9_ orthogonal array and experimental results for different outputs.

Run	Factor levels				Designation	Obtained outputs^*∗*^			Calculated *S/N* ratios for		
	A	B	C	D		BGP yield (mL/gVS)	BMP yield (mL/gVS)	CODS removal (%)	BGP yield	BMP yield	CODS removal
1	33	36	15	6	A1B1C1D1	167.39 ± 16.92	79.58 ± 8.85	68.81 ± 11.96	44.34	37.87	36.35
2	44	36	30	18	A2B1C2D3	240.01 ± 5.25	124.09 ± 6.84	69.85 ± 13.16	47.60	41.83	36.40
3	44	46	0	6	A2B2C3D1	270.99 ± 7.87	126.78 ± 6.74	57.69 ± 3.19	48.65	42.03	35.18
4	33	46	30	11	A1B2C2D2	136.28 ± 3.18	67.76 ± 9.21	−123.25 ± 12.96	42.68	36.35	33.26
5	55	56	30	6	A3B3C2D1	104.02 ± 5.83	61.64 ± 5.35	−81.33 ± 25.72	40.30	35.70	35.89
6	55	46	15	18	A3B2C1D3	86.54 ± 10.15	41.11 ± 6.77	80.57 ± 0.40	38.56	31.86	38.12
7	33	56	0	18	A1B3C3D3	17.07 ± 0.92	9.34 ± 1.73	−263.42 ± 00	24.60	18.96	48.41
8	44	56	15	11	A2B3C1D2	25.55 ± 1.51	13.62 ± 3.16	−91.95 ± 29.89	28.10	21.81	37.81
9	55	36	0	11	A3B1C3D2	140.11 ± 0.31	69.98 ± 4.79	77.44 ± 4.03	42.93	36.83	37.75
Control 1	-	-	-	-	CM:Sludge	302.87 ± 7.71	166.40 ± 5.39	42.57 ± 1.50	-	-	-
Control 2	-	-	-	-	ORWW:Sludge	20.67 ± 2.35	0.00	0.00	-	-	-

^*∗*^Mean ± Stdev.

**Table 4:** *S/N* responses for different outputs.

Outputs	Parameters	Levels			Range	Rank
		Level 1	Level 2	Level 3		
Biogas production	A: ORWW portion in digester (%)	37.21	41.45	40.59	4.24	4
	B: temperature (°C)	44.96	43.29	31.00	13.95	1
	C: sonication time (min)	37.00	43.53	38.73	6.53	3
	D: TS level in digester (%)	44.43	37.90	36.92	7.51	2

Biomethane production	A: ORWW portion in digester (%)	31.06	35.22	34.79	4.16	4
	B: temperature (°C)	38.84	36.75	25.49	13.35	1
	C: sonication time (min)	30.51	37.96	32.61	7.45	3
	D: TS level in digester (%)	38.53	31.66	30.88	7.65	2

CODS removal	A: ORWW portion in digester (%)	39.34	36.46	37.25	2.88	4
	B: temperature (°C)	36.83	35.52	40.70	5.18	2
	C: sonication time (min)	37.43	35.18	40.44	5.26	1
	D: TS level in digester (%)	35.81	36.27	40.98	5.17	3

**Table 5:** Verification experimental results for the A2B1C2D1 formulation.

Number	Cumulative BGP (mL/gVS)	Cumulative BMP (mL/gVS)	CODS stabilization (%)
1	298.23	151.95	70.12
2	275.15	147.21	66.54
3	310.89	156.68	74.00
Average	294.76	151.95	70.22

Predicted by software	306.21	155.25	68.35

**Table 6:** Selection of neurons in the hidden layer.

Number of hidden neurons	Mean square error (MSE)			Correlation coefficient (*R*)			
	Training	Validation	Test	Training	Validation	Test	All
5	315.8403	333.8052	1726.2233	0.9384	0.9589	0.8045	0.9052
10	509.7489	597.2636	871.1606	0.9209	0.9119	0.8124	0.8986
15	366.1776	390.7364	991.7434	0.9317	0.8889	0.8948	0.9169
20	166.2242	882.3070	1253.0458	0.9712	0.9020	0.7213	0.9227
25	181.0968	499.4849	2919.6910	0.9709	0.9287	0.5398	0.8883
30	66.9453	1253.3547	6952.3913	0.9896	0.6727	0.5548	0.8139
50	17.9675	14269.6345	6632.0505	0.9962	0.5130	0.3574	0.6443
16	247.0099	1088.4714	944.7495	0.9557	0.8219	0.8931	0.9175
17	314.0407	519.0463	1295.0456	0.9355	0.9040	0.8693	0.9129
18	187.2529	626.7710	1326.1371	0.9615	0.9039	0.8508	0.9243
19	**202.2901**	**639.2647**	**919.5157**	**0.9667**	**0.9166**	**0.8661**	**0.9348**

## Notations

CM: Chicken manure
ORWW: Oil refinery wastewater
AcoD: Anaerobic codigestion
TS: Total solid
VS: Volatile solid
TOC: Total organic carbon
OM: Organic matter
TN: Total nitrogen
C : N: Carbon : nitrogen ratio
CODS: Chemical oxygen demand solubilization
BGP: Cumulative biogas production
BMP: Cumulative biomethane production
TAN: Total ammonia nitrogen
FAN: Free ammonia nitrogen
ANN: Artificial neural network.

## Additional Points

*Highlights*. (i) Applying Taguchi's L_9_ array to maximize biogas production. (ii) Improving CODS removal of 64.95% of digested substrates. (iii) Developing the 9-19-2 ANN to model the biogas production and biomethane content.

## References

[1] Abouelenien F., Kitamura Y., Nishio N., Nakashimada Y. (2009). Dry anaerobic ammonia-methane production from chicken manure. *Applied Microbiology and Biotechnology*.

[2] Zhang C., Su H., Baeyens J., Tan T. (2014). Reviewing the anaerobic digestion of food waste for biogas production. *Renewable & Sustainable Energy Reviews*.

[3] Rajagopal R., Massé D. I., Singh G. (2013). A critical review on inhibition of anaerobic digestion process by excess ammonia. *Bioresource Technology*.

[4] Zhang Q., Hu J., Lee D.-J. (2016). Biogas from anaerobic digestion processes: Research updates. *Journal of Renewable Energy*.

[5] Siddique M. N. I., Abdul Munaim M. S., Zularisam A. W. (2015). Feasibility analysis of anaerobic co-digestion of activated manure and petrochemical wastewater in Kuantan (Malaysia). *Journal of Cleaner Production*.

[6] Chen J. L., Ortiz R., Steele T. W. J., Stuckey D. C. (2014). Toxicants inhibiting anaerobic digestion: A review. *Biotechnology Advances*.

[7] Abouelenien F., Namba Y., Kosseva M. R., Nishio N., Nakashimada Y. (2014). Enhancement of methane production from co-digestion of chicken manure with agricultural wastes. *Bioresource Technology*.

[8] Diya’uddeen B. H., Daud W. M. A. W., Abdul Aziz A. R. (2011). Treatment technologies for petroleum refinery effluents: a review. *Process Safety and Environmental Protection*.

[9] Haak L., Roy R., Pagilla K. (2016). Toxicity and biogas production potential of refinery waste sludge for anaerobic digestion. *Chemosphere*.

[10] Xi Y., Chang Z., Ye X., Xu R., Du J., Chen G. (2014). Methane production from wheat straw with anaerobic sludge by heme supplementation. *Bioresource Technology*.

[11] Ma S., Wang H., Wang Y., Bu H., Bai J. (2011). Bio-hydrogen production from cornstalk wastes by orthogonal design method. *Journal of Renewable Energy*.

[12] Jacob S., Banerjee R. (2016). Modeling and optimization of anaerobic codigestion of potato waste and aquatic weed by response surface methodology and artificial neural network coupled genetic algorithm. *Bioresource Technology*.

[13] Chen G.-L., Chen G.-B., Li Y.-H., Wu W.-T. (2014). A study of thermal pyrolysis for castor meal using the Taguchi method. *Energy*.

[14] Chan Y. H., Dang K. V., Yusup S., Lim M. T., Zain A. M., Uemura Y. (2014). Studies on catalytic pyrolysis of empty fruit bunch (EFB) using Taguchi's L9 Orthogonal Array. *Journal of the Energy Institute*.

[15] Ghani J. A., Choudhury I. A., Hassan H. H. (2004). Application of Taguchi method in the optimization of end milling parameters. *Journal of Materials Processing Technology*.

[16] Zhen G., Lu X., Kobayashi T., Kumar G., Xu K. (2016). Anaerobic co-digestion on improving methane production from mixed microalgae (Scenedesmus sp., Chlorella sp.) and food waste: Kinetic modeling and synergistic impact evaluation. *Chemical Engineering Journal*.

[17] Nair V. V., Dhar H., Kumar S., Thalla A. K., Mukherjee S., Wong J. W. C. (2016). Artificial neural network based modeling to evaluate methane yield from biogas in a laboratory-scale anaerobic bioreactor. *Bioresource Technology*.

[18] Akbaş H., Bilgen B., Turhan A. M. (2015). An integrated prediction and optimization model of biogas production system at a wastewater treatment facility. *Bioresource Technology*.

[19] Hassan M., Ding W., Bi J., Mehryar E., Talha Z. A. A., Huang H. (2016). Methane enhancement through oxidative cleavage and alkali solubilization pre-treatments for corn stover with anaerobic activated sludge. *Bioresource Technology*.

[20] APHA (2006). *Standard Methods for the Examination of Water and Wastewater*.

[21] Dhussa A. K., Sambi S. S., Kumar S., Kumar S., Kumar S. (2014). Nonlinear Autoregressive Exogenous modeling of a large anaerobic digester producing biogas from cattle waste. *Bioresource Technology*.

[22] Aslanzadeh S. (2014). *Pretreatment of Cellulosic Waste and High Rate Biogas Production*.

[23] Kwietniewska E., Tys J. (2014). Process characteristics, inhibition factors and methane yields of anaerobic digestion process, with particular focus on microalgal biomass fermentation. *Renewable & Sustainable Energy Reviews*.

[24] Wang H., Fotidis I. A., Angelidaki I. (2016). Ammonia-LCFA synergetic co-inhibition effect in manure-based continuous biomethanation process. *Bioresource Technology*.

[25] Ahn H. K., Smith M. C., Kondrad S. L., White J. W. (2010). Evaluation of biogas production potential by dry anaerobic digestion of switchgrass-animal manure mixtures. *Applied Biochemistry and Biotechnology*.

